# Drought prediction based on an improved VMD-OS-QR-ELM model

**DOI:** 10.1371/journal.pone.0262329

**Published:** 2022-01-06

**Authors:** Yang Liu, Li Hu Wang, Li Bo Yang, Xue Mei Liu

**Affiliations:** School of Information Engineering, North China University of Water Resources and Electric Power, Zhengzhou, Henan, China; TDTU: Ton Duc Thang University, VIET NAM

## Abstract

To overcome the low accuracy, poor reliability, and delay in the current drought prediction models, we propose a new extreme learning machine (ELM) based on an improved variational mode decomposition (VMD). The model first redefines the output of the hidden layer of the ELM model with orthogonal triangular matrix decomposition (QR) to construct an orthogonal triangular ELM (QR-ELM), and then introduces an online sequence learning mechanism (OS) into the QR-ELM to construct an online sequence OR-ELM (OS-QR-ELM), which effectively improves the efficiency of the ELM model. The mutual information extension method was then used to extend both ends of the original signal to improve the VMD end effect. Finally, VMD and OS-QR-ELM were combined to construct a drought prediction method based on the VMD-OS-QR-ELM. The reliability and accuracy of the VMD-OS-QR-ELM model were improved by 86.19% and 93.20%, respectively, compared with those of the support vector regression model combined with empirical mode decomposition. Furthermore, the calculation efficiency of the OS-QR-ELM model was increased by 88.65% and 85.32% compared with that of the ELM and QR-ELM models, respectively.

## Introduction

Currently, the water cycle is affected by several factors, and there is no clear distinction between the occurrence, development, and end of drought, which makes it difficult to accurately determine the duration of drought [1]. Therefore, accurate and timely drought forecasting is still a challenge in drought resistance and disaster mitigation research. Thus, forecasting drought, identifying the occurrence of drought several weeks or months in advance, and predicting the development and retreat process of drought are important to formulate scientific and effective drought response strategies in a timely manner and to reduce the losses caused by disasters [2].

To forecast drought within few days, months, or even years, hydrological forecasters have different methods to choose from. These mainly include hydrological models based on mechanism and statistical models based on data. Mechanism-driven models include those that integrate rainfall, soil moisture, and vegetation dynamics, and they use soil moisture models to track soil moisture, normalise difference in vegetation index, and finally forecast drought [3]. According to the meteorological data from weather stations, the Palmer Drought Severity Index has been used to forecast drought. The results indicate that compared with historical periods, drought periods will increase in the next 30 years [4]. The distributed basin-scale model has been used to study the effect of climate change on the hydrology of basins; the results showed that river flow and groundwater recharge have reduced the most, and that the degree of drought has increased [5]. However, mechanism-driven models often have the following problems: numerous external interference factors and poor understanding of the mechanism [6, 7]. According to their structure, data-driven models can be divided into monomer and hybrid models. The former uses indexes of precipitation, vegetation condition, temperature, and soil as inputs for artificial neural network (ANN) models of drought prediction and assessment. A previous study showed that the accuracy of the ANN models reached 92% [8]. Support vector regression (SVR) models are used to predict the standardised precipitation evapotranspiration index for drought assessment. A previous study showed that the support vector machine models perform well in drought prediction [9]. The Multilayer Perceptron Neural Network (MLPNN) is used to predict the standardised precipitation index (SPI), and the root-mean-square-error (RMSE), Nash efficiency index (E_ns_), correlation coefficient, and Wilmot index are used as evaluation criteria. The MLPNN model is better than other models in predicting the SPI [10]. However, a single model often has a poor generalisation ability and low prediction accuracy; mixed models can effectively overcome such problems [11–14]. For example, a drought prediction model, Wavelet-ARIMA-ANN, combines the advantages of wavelet transform, autoregressive integrated moving average (ARIMA), and ANN models. A previous study reported that the overall correlation coefficient (R) of the ANN model was 0.423, but the R-value of the Wavelet-ARIMA-ANN model was reduced to 0.415 [15]. The drought prediction model EMD-ANFIS was constructed by combining empirical mode decomposition (EMD) and adaptive neuro-fuzzy inference system (ANFIS) models. When the prediction step length was 3 and 6 months, the E_ns_ of ANFIS was 0.52 and 0.17, respectively, whereas that of EMD-ANFIS was 0.81 and 0.77, respectively [16]. However, the EMD methods often have issues with modal aliasing [17] and end effects [18]. The variational modal decomposition (VMD) method can effectively solve the problem of modal aliasing in EMD [19] and has been applied in timing predictions [20]. By combining VMD, particle swarm optimisation algorithm (IPSO), and deep confidence network (DBN), a VMD-DBN-IPSO time series prediction model has been constructed, with mean absolute error (MAE), RMSE, and E_ns_ used as the evaluation criteria. The VMD-DBN-IPSO model achieved the best performance in the training and testing phases and presented high stability and representativeness. The E_ns_ coefficient was maintained above 0.8, and the peak flow prediction error was within 20% [21]. However, although the VMD method eliminates the problem of modal aliasing, the end effect still exists [22–24].

Based on the above discussion, in this study, we propose a new VMD-based extreme learning machine (ELM) model and applied it to regional drought prediction. Its main contributions are as follows:

- First, the output of the ELM hidden layer is redefined by orthogonal triangular matrix decomposition (QR), and an online sequence learning mechanism (OS) is introduced to construct the OS-QR-ELM prediction model, which can effectively improve the calculation efficiency of the ELM model.
- Based on the mutual information extension method, both ends of the original signal are then extended to solve the VMD end effect.
- Finally, the improved VMD and OS-QR-ELM are combined, and parallel computing ideas are introduced to construct a drought prediction model based on VMD-OS-QR-ELM. This can effectively improve the prediction accuracy and credibility of the model.

The optimal calculation results based on the VMD-OS-QR-ELM indicate that this method can effectively improve the ability and efficiency of regional disaster prevention and relief.

## Correlation theory

### OS-QR-ELM

**Fig 1:**
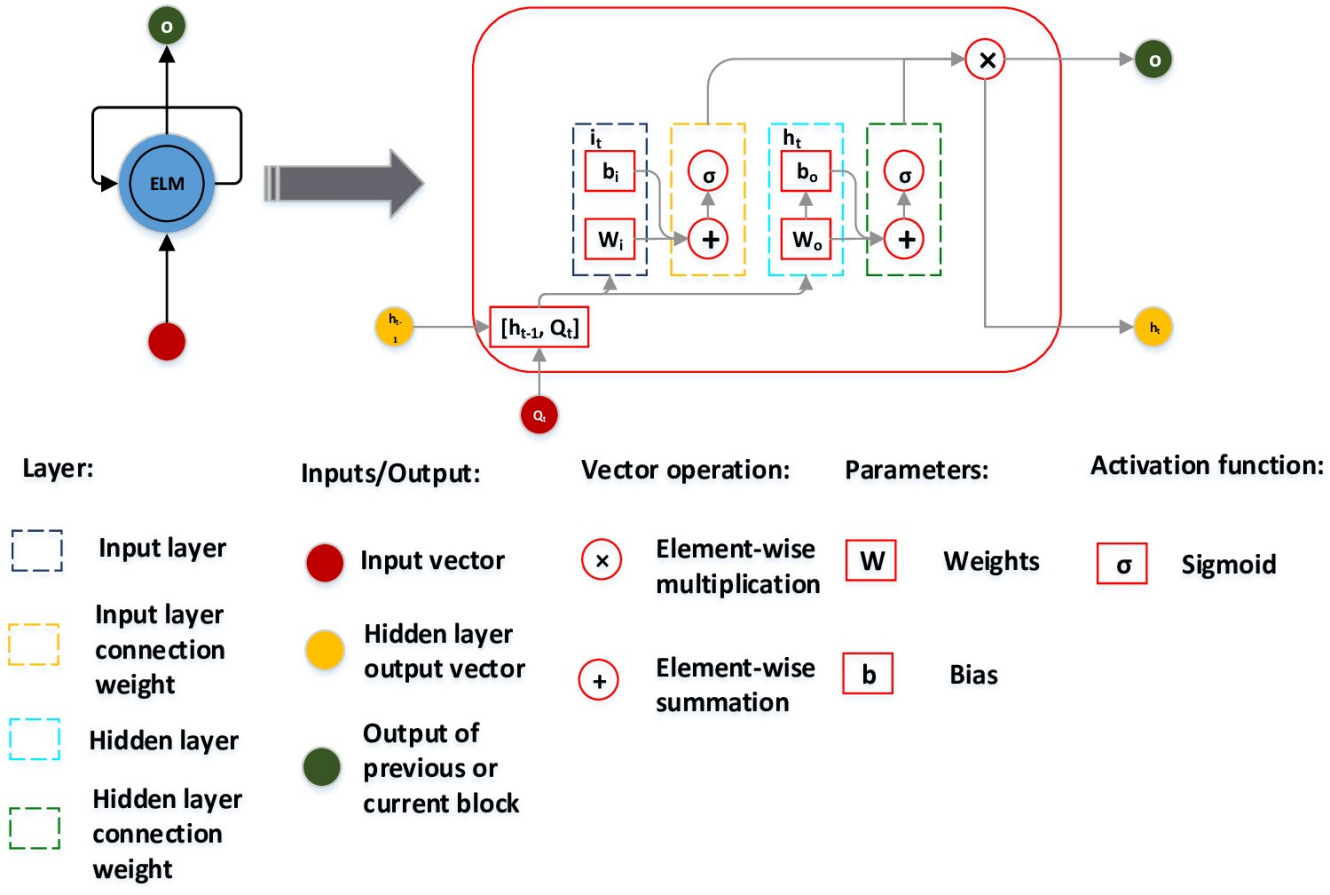
Extreme learning machine topology.

An ELM is a machine learning method based on a feedforward neural network. Unlike ANNs, the weights of the nodes in the hidden layer of the ELM model are artificially assigned and do not require updating [25]. QR matrix decomposition is an effective method of solving all eigenvalues of general matrices and is widely used in matrix generalised inverse calculation and least-squares problem solving [26]. The online sequence learning mechanism can effectively improve the computational efficiency of the model while ensuring its generalisation ability. Fig 1 shows the topological structure of an ELM, and its basic implementation is as follows.

The input, hidden, and output layers are defined as *Q*, *H*, and *O*, respectively. The input sample *Q*_*t*_ is defined as:

$$Q_{t}={\left[\begin{array}{cccc} Q_{1} & Q_{2} & \ldots & Q_{n} \end{array}\right]}^{T}$$ (1)

A neural network with *m* hidden layer nodes can be defined as:

$$\sum_{i=1}^{m}\beta \sigma \left(w_{i}x_{j}+b_{i}\right)=O_{j},j=1,2,\ldots n$$ (2)

where, *σ*(*x*) is the activation function, *w*_*i*_ is the input weight of the *i* hidden layer unit, *b*_*i*_ is the bias of the *i* hidden layer unit, *β*_*i*_ = [*β*_1_   *β*_2_ … *β*_*n*_]^*T*^ is the output weight of the *i* hidden layer unit, and *w*_*i*_*x*_*j*_ represents the inner product of *w*_*i*_ and *x*_*j*_. The basic gradient-learning-based algorithm adjusts the parameters in an iterative manner; however, in the ELM algorithm, once the input weight *w*_*i*_ and hidden layer bias *b*_*i*_ are randomly determined, the hidden layer output matrix *H* is uniquely determined, and training a single-layer neural network can be transformed into a linear equation, as follows:

Hβ=T (3)

The output weight can be uniquely determined as:

$$\beta =H^{+}T$$ (4)

where, *H*^+^ is the plus generalised inverse of the matrix *H* Moore-Penrose), and the smallest and unique norm of *β* can be obtained [27–31].

The traditional ELM method generally uses singular value decomposition (SVD) to solve the hidden layer output, and the SVD method can be defined as follows:

$$H=U\sum V^{T}$$ (5)

where, *U* and *V* are orthogonal unitary matrix $\sum \left(\begin{array}{cc} \Delta & 0\\ 0 & 0 \end{array}\right)$, and *Δ* is the invertible diagonal matrix. In this study, we used QR decomposition to redefine the output of the ELM hidden layer. The QR method has a higher efficiency and a simpler calculation process than the SVD method, which can effectively improve the calculation efficiency of ELM. The basic form of QR decomposition can be defined as follows:

A=QR (6)

where, *Q* is an orthogonal matrix and *R* is an upper triangular matrix. According to the related partitioned matrix theory [32],

$${\left[\begin{array}{cc} B & D\\ 0 & C \end{array}\right]}^{-1}=\left[\begin{array}{cc} B^{-1} & -B^{-1}{\text{DC}}^{-1}\\ 0 & C^{-1} \end{array}\right]$$ (7)

where, *B* and *C* are reversible. Hence,

$$\begin{array}{c} R_{l+1}^{-1}={\left[\begin{array}{cc} R_{l} & r_{l+1}\\ 0 & r_{l+1,l+1} \end{array}\right]}^{-1}\\ =\left[\begin{array}{cc} R_{l}^{-1} & -R_{l}^{-1}r_{l+1}r_{l+1,l+1}^{-1}\\ 0 & r_{l+1,l+1}^{-1} \end{array}\right] \end{array}$$ (8)

The QR-ELM hidden layer output can then be redefined as follows:

$$\begin{array}{c} H^{+}T=R_{l+1}^{-1}Q_{l+1}^{T}T\\ =\left[\begin{array}{cc} R_{l}^{-1} & -R_{l}^{-1}r_{l+1}r_{l+1,l+1}^{-1}\\ 0 & r_{l+1,l+1}^{-1} \end{array}\right]\left[\begin{array}{c} Q_{l}^{T}\\ q_{l+1}^{T} \end{array}\right]T\\ =\left[\begin{array}{c} f_{l}-R_{l}^{-1}r_{l+1}f_{l+1}^{T}\\ f_{l+1}^{T} \end{array}\right] \end{array}$$ (9)

On this basis, the online sequence learning mechanism was introduced into the QR-ELM model to build the OS-QR-ELM. The learning process of the output weight in the OS-QR-ELM model was divided into two parts: the initial stage, where the initial output weight is obtained through a small number of samples, and the online learning stage, which uses a single sample or sample data block to update the output weight of the single hidden layer feedforward neural network learned in the initial stage. For OS-QR-ELM, *β*_*os*_ is expressed as a function of *β*, *H*, and *T*, defined as follows:

$$\begin{array}{c} \beta_{os}=K_{1}^{-1}\left[\begin{array}{c} H_{0}\\ H_{1} \end{array}\right]\left[\begin{array}{c} T_{0}\\ T_{1} \end{array}\right]\\ =K_{1}^{-1}\left(K_{1}\beta -H_{1}^{T}H_{1}\beta +H_{1}^{T}H_{1}\right)\\ =\beta +K_{1}^{-1}H_{1}^{T}\left(T_{1}-H_{1}\beta \right) \end{array}$$ (10)

Among them, *K*_1_ is defined as follows:

$$K_{1}=\left[\begin{array}{cc} H_{0}^{T} & H_{0}^{T} \end{array}\right]\left[\begin{array}{c} H_{0}\\ H_{1} \end{array}\right]$$ (11)

### VMD

**Fig 2:**
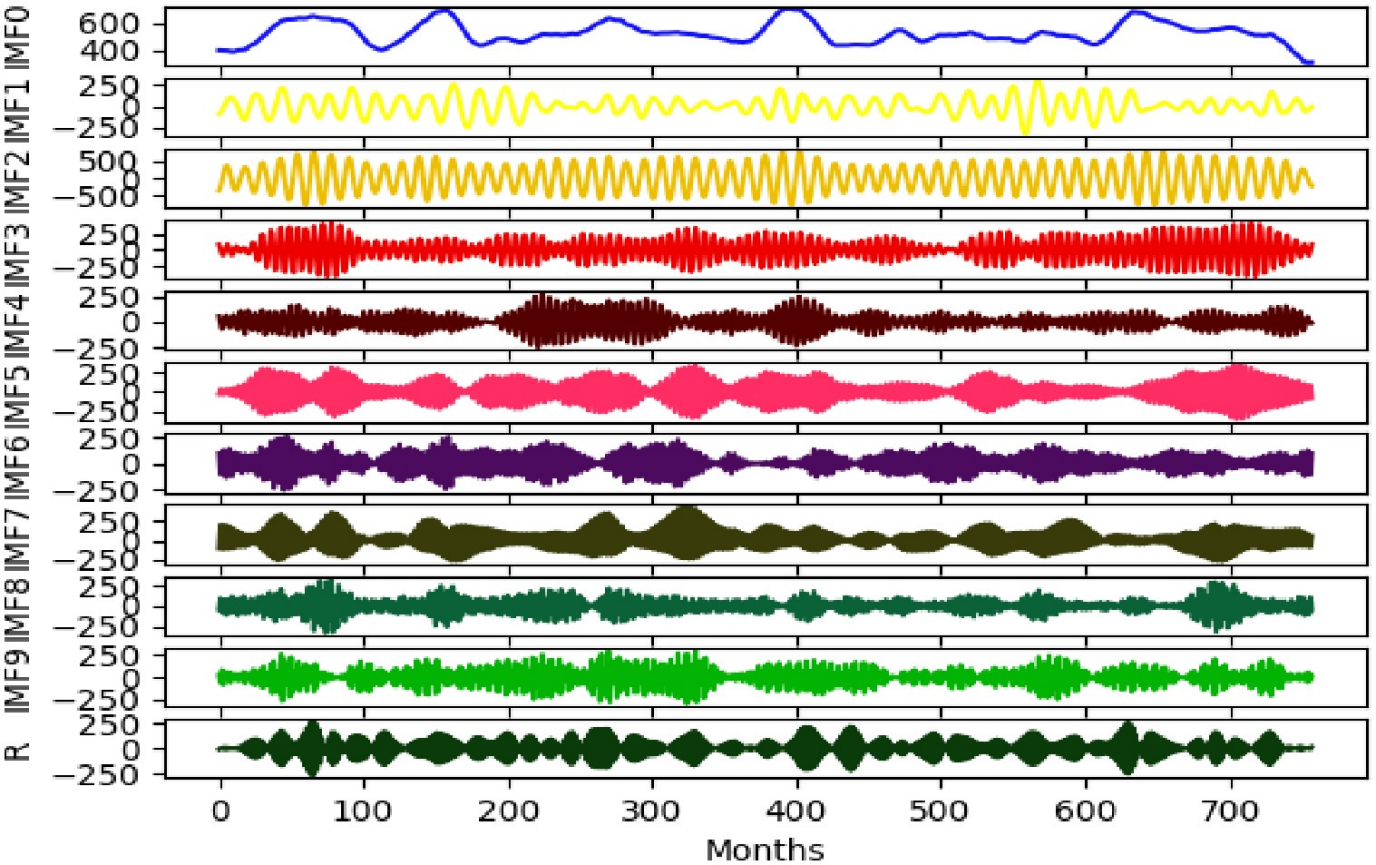
VMD results of drought time series data.

VMD is a signal decomposition estimation method [33] that determines the frequency centre and bandwidth of each component by iteratively searching for the optimal solution of the variational model in the process of obtaining the decomposed components to adaptively achieve the frequency domain division of the signal and effective separation of the components [34, 35]. Fig 2 shows the decomposition effect of VMD, and it is realised as follows.

A given sample *X* is decomposed into k eigenmode components (IMF) with the same centre frequency while ensuring that the sum of the estimated bandwidth of each IMF is the smallest. The corresponding constraint variational expression is defined as follows:

$$\underset{\left\{u_{k}\right\},\left\{\omega_{k}\right\}}{\text{min}}\left\{{\sum_{k=1}^{K}\left\|\partial_{t}\left[\left(\delta \left(t\right)+\frac{j}{\pi t}\right)⊗u_{k}\left(t\right)\right]e^{-j\omega_{k}t}\right\|}_{2}^{2}\right\},s\text{.}t\text{.}\left\{\sum_{k=1}^{K}u_{k}=X\right\}$$ (12)

where, {*u*_*k*_} = {*u*_1_, *u*_2_, …, *u*_*k*_} is the *k* IMF obtained by decomposition, {*ω*_*k*_} = {*ω*_1_, *ω*_2_, …, *ω*_*k*_} is the centre frequency of the component, ⊗ is the convolution calculation, *K* is the total number of modal functions, *δ*(*t*) is the Dirac distribution, and $e^{-{j\omega }_{k}t}$ is the centre frequency of the modal function on the complex plane, with *k* as the centre frequency of the modal function.

Eq (12) is solved, the Lagrange multiplication operator *λ* is introduced, the constrained variational problem is transformed into an unconstrained variational problem, and the augmented Lagrange expression is defined as follows:

$$\begin{array}{c} L\left(\left\{u_{k}\right\},\left\{\omega_{k}\right\},\lambda \right)={\left\|f_{\text{Runoff}}\left(t\right)-\sum_{k}u_{k}\left(t\right)\right\|}_{2}^{2}\\ +\alpha {\sum_{k}\left\|\partial_{t}\left[\left(\delta \left(t\right)+\frac{j}{\pi t}\right)*u_{k}\left(t\right)\right]e^{-j\omega_{k}t}\right\|}_{2}^{2}+\langle \lambda \left(t\right),f_{\text{Runoff}}\left(t\right)-\sum_{k}u_{k}\left(t\right)\rangle \end{array}$$ (13)

The iterative terms {*u*_*k*_}, {*ω*_*k*_}, and use the alternating direction multiplier method to obtain the saddle point of the augmented Lagrangian expression through iterative updating. Among them, the alternating direction multiplier is a computational framework for solving convex optimisation problems with a separable structure and is generally used to solve equation optimisation problems. Compared with other methods, the alternate direction multiplier has advantages of high processing speed and good convergence performance [36–38].

## Construction of drought forecasting methods

### End effect improvement scheme based on mutual information extension

VMD should undergo multiple ‘screenings’ to obtain IMF during the decomposition process. As both ends of the signal cannot be at the maximum or minimum value simultaneously, the IMF will diverge at both ends of the screening process sequence and gradually inwards, thus affecting the VMD; this is the end effect of VMD. Boundary extension methods such as the endpoint mirror method, extreme value extension method, and polynomial fitting method are usually used to solve the boundary effect of modal decomposition. Compared with other methods, the extreme value extension method comprehensively considers the influence of changes in the end extreme value and the size of the internal extreme value on the original sequence, and has the advantages of strong adaptability, long effective expansion distance, and high expansion speed. This study adopts the extreme value extension method based on mutual information criterion, and its basic realisation is as follows.

1. For a sample *X* of a given length *N*, it is necessary to obtain the maximum value sequence and minimum value sequence of *X*, but it is difficult to determine whether the end point is a maximum value or a minimum value. Therefore, the maximum value sequence and minimum value sequence are defined as *T*_max_ = {*t*_*p*1_ *t*_*p*2_ … *t*_pm_} and *T*_min_ = {*t*_*q*1_ *t*_*q*2_ … *t*_ql_}, and their lengths are defined as *M* and *L*, respectively.
2. When *t*_*p*1_ < *t*_*q*1_, then the intercept *X*(*t*_1_) ~ *X*(*t*_*p*1_) band is the wavelet to be matched, defined as *S*_1_; in *X*, *X*(*t*_pj_), *j* ∈ [*i* + 1, *m*] is used as *X*(*t*_pi_). The corresponding points are successively intercepted as the wavelet to be matched with the waveband *S*_*j*_ of the same length as *S*_1_.
3. The mutual information value *I*_*j*_ of *S*_1_ and *S*_*j*_ is calculated, the wavelet $S_{j_{\text{best}}}$ with the largest mutual information is considered as the best matching band of *S*_1_, and then the same length band before $X_{j_{\text{best}}}$ is selected to extend to the left of *X*, where *I*_*j*_ is defined as follows:
$$I_{j}\left(S_{1},S_{j}\right)=H\left(S_{j}\right)-H\left(S_{j}|S_{1}\right)$$ (14)
  Among them, *H*(*S*_*j*_) is the entropy of *S*_*j*_ and *H*(*S*_*j*_|*S*_1_) is the conditional entropy of *S*_*j*_ when *S*_1_ is known. The stronger the correlation between *S*_1_ and *S*_*j*_, the smaller the conditional entropy *S*_*j*_, and the larger the mutual information *I*_*j*_.
4. When *t*_*p*1_ ≥ *t*_*q*1_, the maximum value in step 2 is replaced with the minimum value for processing to complete the left boundary extension of the signal.
5. The same method is used to extend the right boundary of the signal. After completing the boundary extension, VMD is performed on the original signal and the bands corresponding to the original signal position and the same length in each component are intercepted to obtain the final decomposition result.

### Drought forecast model based on improved VMD-OS-QR-ELM

**Fig 3:**
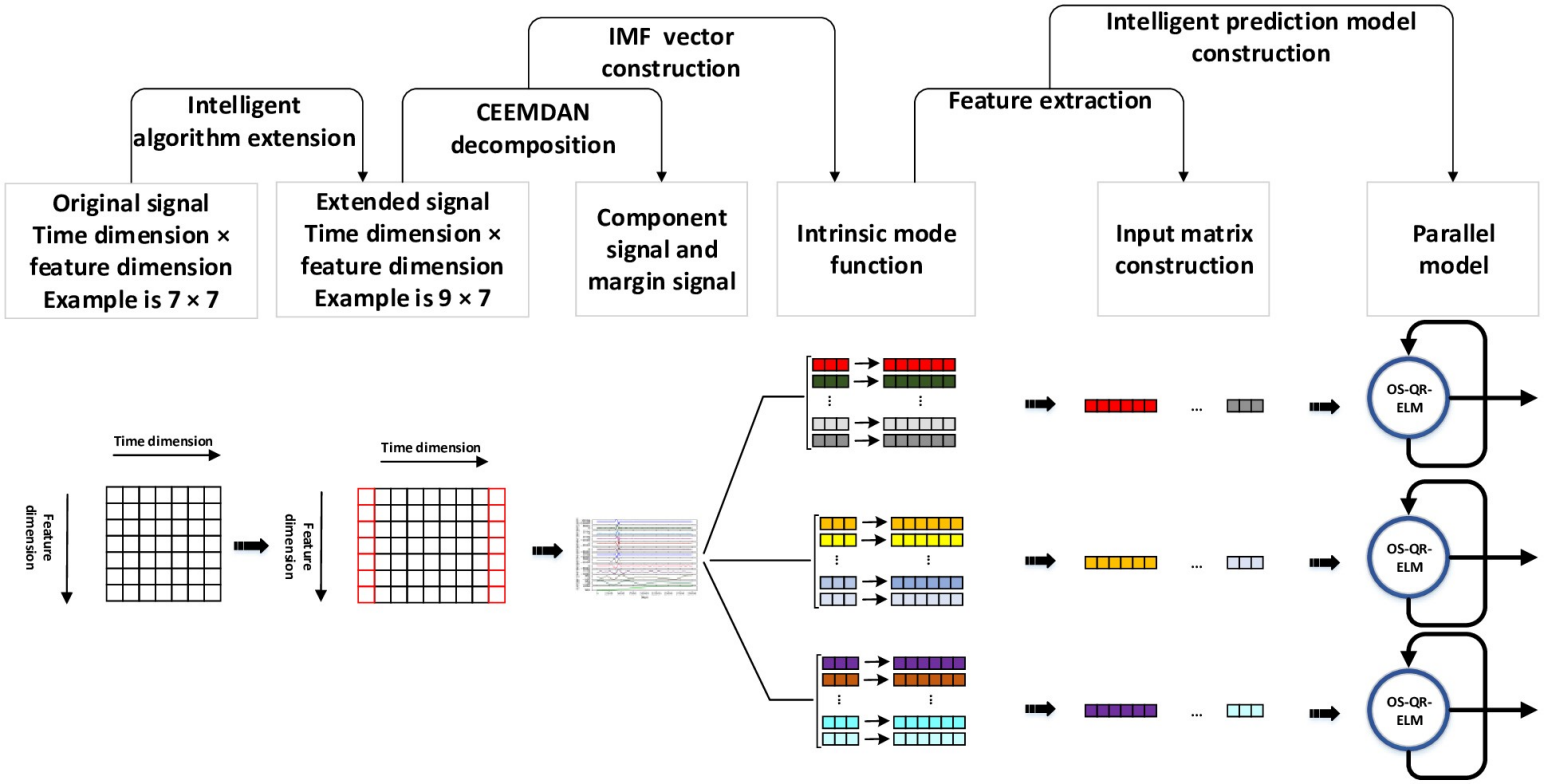
Structure flowchart of the parallel VMD-OS-QR-ELM.

By combining the improved VMD and OS-QR-ELM models, a new enhanced ELM drought prediction model (VMD-OS-QR-ELM model) was developed. Fig 3 shows the basic process of the model, and its basic implementation is described below.

1. Using the monthly scale meteorological data from Anyang, Xinyang, Zhumadian, and Zhengzhou in Henan Province from 1951 to 2021 as the research objects, we predicted the future drought level of some cities in the province. An accurate and reliable drought forecast model is important for urban development in Henan Province, which has a large population and is an agricultural province. In this study, the temperature and rainfall data obtained were first subjected to processes such as null filtering, interpolation, and deduplication. Thereafter, the filtered sample data (defined as X*) were normalised. The normalisation equation is as follows:
$$X^{*}=\frac{X-\text{min}\left(X\right)}{\text{max}\left(X\right)-\text{min}\left(X\right)}$$ (15)
  where, max(*X*) is the maximum value of the sample and min(*X*) is the minimum value of the sample.
2. The filtered and normalised sample data are first extended by the boundary, and the extended sample is then used to initialise and optimise the VMD-related parameters, which mainly include the penalty factor, noise tolerance, mode number, and initialisation centre frequency. The optimisation equation is as follows:
$$J\left(\theta \right)=\frac{1}{2}\overset{m}{\underset{i=1}{\sum }}h_{\theta }\left(x^{i}\right)-y^{i},\;\underset{\theta }{\text{min}}J_{\theta }$$ (16)
  where, h(x) represents the reconstruction data after VMD decomposition and y represents the true value. Finally, the balance parameters, noise tolerance, number of modes, and initial centre frequency were set as 56.0, 0, 11, and 1, respectively. After VMD decomposition, the boundary position corresponding to the original signal was intercepted to obtain the final decomposition result. The decomposed IMFs were randomly divided into k parts, one of which was selected as the test set, and the remaining k − 1 parts were used as the training set.
3. First the OS-QR-ELM model is initialised and trained using the training set; the test set is then input into the model to make future predictions. According to Eq 16, the number of hidden layer nodes and the regularisation coefficient of OS-QR-ELM were set as 20 and 2, respectively.
4. The predicted values of temperature and rainfall are used as inputs, and the de Martonne drought index is used as the drought grade classification standard to establish a drought risk assessment system. Compared with other drought indexes, the de Martonne drought index has a calculation process with the advantages of simplicity and strong applicability, and its definition is as follows:
$$I_{\text{dm}}=\frac{\text{12}R}{T+\text{10}}$$ (17)
  where, *R* represents monthly precipitation and *T* represents monthly average temperature. A de Martonne index value less than 30 indicates the occurrence of drought. An index value between 10 and 30 indicates moderate drought, and a value less than 10 indicates severe drought.

### Model evaluation

E_ns_, MAE, relative error (RE), and Run-Time were considered as the evaluation criteria to evaluate the reliability, stability, accuracy, and execution efficiency of the algorithm. The E_ns_ Nash efficiency coefficient was used to evaluate the credibility and stability of the prediction model. E_ns_ ranges from negative infinity to 1, and when it is close to 1, the model has a good quality and high credibility. When E_ns_ is close to 0, the simulation result is close to the average level of the observed value (i.e. the overall result is credible); however, the process simulation error is large. When E_ns_ is significantly < 0, the model is not credible. The RE and average MAE were used to evaluate the real-time and overall errors, respectively.

## Results

In this study, we used the monthly average temperature and monthly average precipitation from 1951 to 2021 in Anyang, Xinyang, Zhumadian, and Zhengzhou in Henan Province as data objects; OS-QR-ELM, QR-ELM, least squares support vector regression (LSSVR) [39, 40], EMD-SVR, and multilayer perceptron (MLP) [41] were used as the comparison algorithms for VMD-OS-QR-ELM, and E_ns_, MAE, RE, and operating time were used as the evaluation criteria. A comprehensive evaluation was carried out, and the results are as follows.

**Fig 4:**
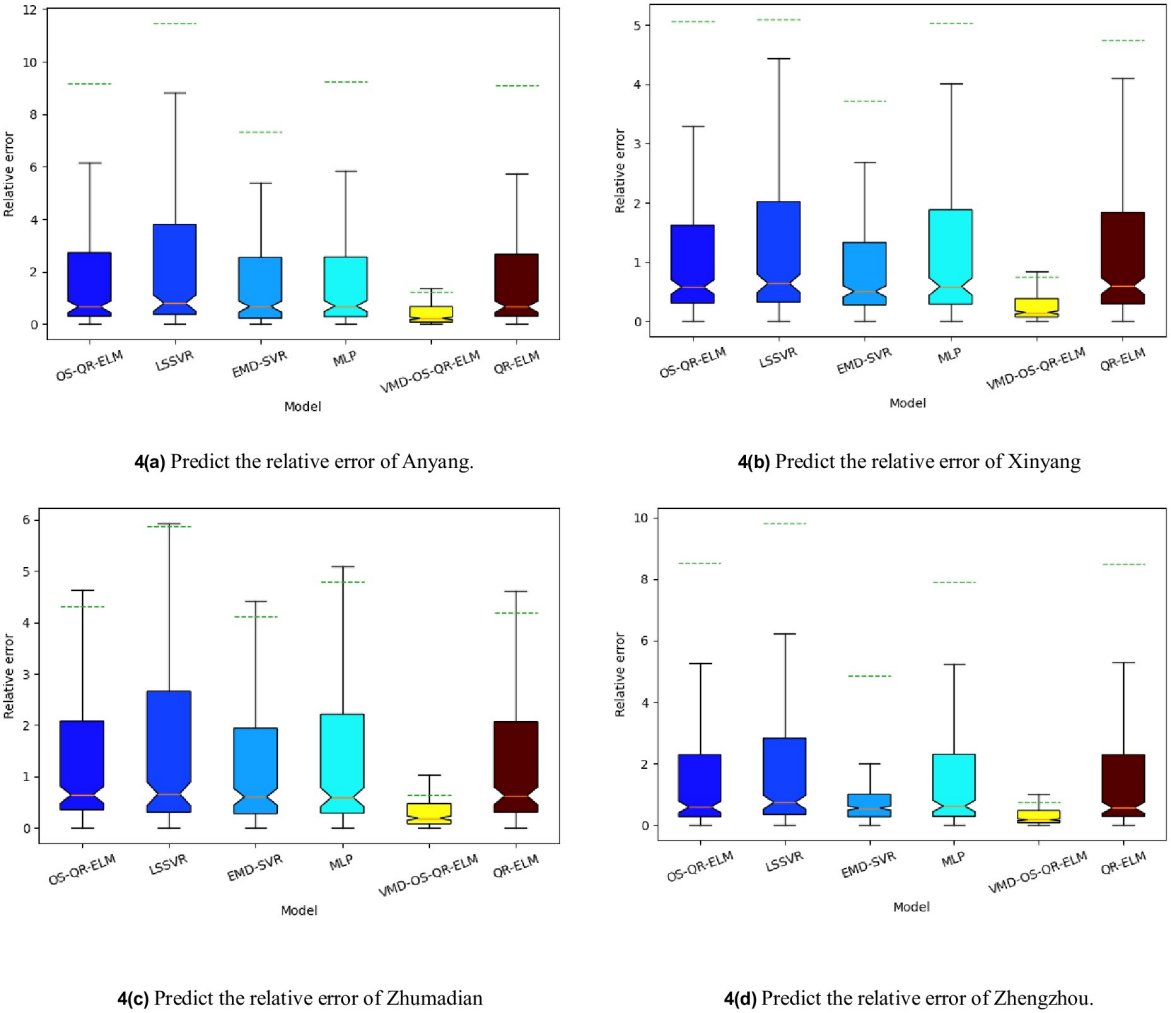
Relative errors of different models and cities.

Fig 4 shows the RE level of each model. Compared with QR-ELM, SVR, and VMD-SVR, VMD-OS-QR-ELM uses a modal decomposition process, and thus, has obvious advantages in processing non-stationary and nonlinear data. Although the EMD-SVR is better than the LSSVR model, the EMD method is prone to modal aliasing during the decomposition process, because of which the error level of the EMD-SVR is higher than that of the VMD-OS-QR-ELM model.

Table 1 shows the evaluation index results of each model, Table 2 shows the calculation time for each model, and Table 3 shows the IMF reconstruction error after mutual information extension and including extreme unknown endpoints. Compared with the EMD-SVR model, VMD-OS-QR-ELM can complete adaptive decomposition of the signal according to the frequency domain characteristics of the original signal, which results in higher robustness and generalisation ability of the VMD-OS-QR-ELM model. According to the details in Table 1, when VMD-OS-QR-ELM was compared with EMD-SVR, Ens increased by 86.19% and MAE decreased by 93.20%. According to the information in Table 2, compared with the ELM and OR-ELM models, OS-QR-ELM uses a highly efficient QR decomposition scheme and introduces an online learning mechanism, which makes OS-QR-ELM more efficient than the ELM and QR-ELM calculations The efficiency increased by 88.65% and 85.32%, respectively. According to the information in Table 3, after extending the original signal by mutual information, the end effect is weakened, and IMF has more physical meaning. Therefore, the reconstruction error of VMD is reduced by 43.51%.

**Table 1:** Comparison of the numerical results for various evaluation indicators.

Evaluation index	Model	Anyang	Xinyang	Zhumadian	Zhengzhou
**E**_**ns**_	VMD-OS-QR-ELM	0.998	0.997	0.997	0.998
	OS-QR-ELM	0.210	-0.020	0.124	0.257
	QR-ELM	0.225	0.146	0.158	0.254
	LSSVR	0.054	0.064	0.070	0.095
	EMD-SVR	0.432	0.462	0.437	0.536
	MLP	0.216	0.156	0.175	0.268
**MAE**	VMD-OS-QR-ELM	0.218	0.351	0.328	0.205
	OS-QR-ELM	3.554	5.641	5.350	3.522
	QR-ELM	3.507	5.378	5.093	3.511
	LSSVR	4.129	5.855	5.475	4.066
	EMD-SVR	3.430	4.624	4.587	3.014
	MLP	3.505	5.470	5.098	3.537

**Table 2:** Comparison of the computing time for various models.

Model	Calculation and prediction time (s)
**ELM**	0.141
**QR-ELM**	0.109
**OS-QR-ELM**	0.016

**Table 3:** IMF reconstruction error in different scenarios.

Scenes	Anyang	Nanyang	Zhumadian	Zhengzhou
**Extreme value extension method**	1.180	0.443	0.804	2.333
**Endpoint extreme value unknown**	5.443	2.124	2.076	4.130

## Conclusions

With the aim to overcome the problems of low prediction accuracy, poor reliability, and low calculation efficiency in drought prediction, a new drought prediction model based on VMD-OS-QR-ELM was proposed. First, the QR method was used to redefine the output of the ELM hidden layer, and an online sequence learning mechanism was introduced to construct an OS-QR-ELM prediction model. The mutual information extension method was then used to improve the end effect of VMD, and the improved VMD method was combined with the OS-QR-ELM model to construct the VMD-OS-QR-ELM drought prediction model. The results indicated that when compared with that of ELM and QR-ELM, the calculation efficiency of OS-QR-ELM was increased by 88.65% and 85.32%, respectively; compared with those of EMD-SVR, the reliability and accuracy of the VMD-OS-QR-ELM model were increased by 86.19% and 93.20%, respectively. Furthermore, the VMD method introduced in this study increased the decomposition and synthesis process, which indirectly reduced the computational efficiency of the model and increased its computational cost. Although the introduced parallel computing idea can effectively improve the computational efficiency of the serial model, it still cannot solve the problem fundamentally. In future research on VMD-OS-QR-ELM, if the computational efficiency problem of the model can be overcome, its overall performance can be improved.
